# Point-of-care bedside ultrasound examination for the exclusion of clinically significant ankle and fifth metatarsal bone fractures; a single blinded prospective diagnostic cohort study

**DOI:** 10.1186/s13047-020-00387-y

**Published:** 2020-05-07

**Authors:** Aniek Crombach, Nasim Azizi, Heleen Lameijer, Mostafa El Moumni, Jan C. ter Maaten

**Affiliations:** 1 Department of Emergency Medicine, University Medical Centre Groningen, University of Groningen, Hanzeplein 1, 9700 RB Groningen, the Netherlands; 2 Department of Emergency Medicine, Medical Centre Leeuwarden, Leeuwarden, the Netherlands; 3 Department of Surgery, University Medical Centre Groningen, University of Groningen, Groningen, the Netherlands; 4 Department of Internal Medicine, University Medical Centre Groningen, University of Groningen, Groningen, the Netherlands

**Keywords:** Point-of-care bedside ultrasound, PoCUS, Emergency ultrasound, Bedside ultrasound, Emergency department, Ankle fractures, Foot fractures

## Abstract

**Objective:**

The aim of this study was to assess the diagnostic value of point-of-care bedside ultrasound (PoCUS) as in usual clinical practice in suspected ankle and fifth metatarsal bone fractures, compared to the standard of radiographic imaging.

**Methods:**

This prospective study included patients *≥*17 years presenting to the Emergency Department with ankle trauma and positive Ottawa Ankle Rules. All patients underwent PoCUS of the ankle by a (resident) emergency physician, the images were assessed by an ultrasound expert. Both were blinded for the medical history and clinical findings of the patients. Radiography of the ankle followed, evaluated by a radiologist blinded from the PoCUS findings. Primary outcome measures were sensitivity and specificity of PoCUS.

**Results:**

A total of 242 patients were included, with 35 (22%) clinically significant (non-avulsion) fractures observed with radiography. The sensitivity of PoCUS in detecting clinically significant fractures by all sonographers was 80.0% (95% Confidence Interval (CI) 63.0 to 91.6%), specificity 90.3% (95% CI 83.7 to 94.9%), positive predictive value 70.0% (95% CI 57.0 to 80.3%) and the negative predictive value 94.1% (95% CI 89.1 to 96.9%). The sensitivity of PoCUS in detecting clinically significant fractures by the expert was 82.8% (95% CI 66.3 to 93.4%), specificity 99.2% (95% CI 95.5 to 99.9%), positive predictive value 96.7% (95% CI 80.3 to 99.5%) and the negative predictive value 95.3% (95% CI 91.0 to 98.2%).

**Conclusion:**

PoCUS combined with the OAR has a good diagnostic value in usual clinical practice in the assessment of suspected ankle and fifth metatarsal bone fractures compared to radiographic imaging. More experience with PoCUS will improve the diagnostic value.

**Trial registration:**

Registered in the local Research Register, study number 201500597.

## Background

Foot and ankle injuries are one of the most frequent reasons to visit the Emergency Department (ED), but the diagnostic instruments used are highly inefficient [1]. Plain radiographs are commonly used to diagnose a suspected fracture in foot and ankle trauma. The relative low probability of fractures causes frequent unnecessary radiation exposure, together with burdening of the patient and costs, time, and crowding in the ED. [2]

To reduce the use of radiography for the assessment of ankle injuries the Ottawa Ankle Rules (OAR) were developed [2–4]. The OAR has a high sensitivity but are low in specificity, leading again to a lesser but still significant amount of unnecessary radiation exposure in these patients [2, 4].

A few studies have assessed the use of point-of-care bedside ultrasound (PoCUS) in diagnostics of foot and ankle injuries specifically, showing a sensitivity ranging from 87.3 to 100% and specificity ranging from 90.1 to 99.1%. These studies show that when PoCUS is used in OAR positive patients, there can be an approximately 80% reduction of radiological assessment. The studies on the matter are all subjective to bias regarding PoCUS, because of unblinded designs, limited amount of and selected sonographers and non-consecutive inclusion [2, 5].

In this prospective single blinded cohort study we aim to assess the diagnostic value of PoCUS in suspected fifth metatarsal bone and ankle fractures compared to the use of radiographic imaging.

## Methods

### Study desig

This was a single blinded prospective diagnostic cohort study, conducted between August 2015 and December 2017. The goal was to compare PoCUS to the reference standard of radiographic imaging. Approval by the local ethics committee was obtained and patients informed consent documented. The Standards for Reporting Diagnostic Accuracy (STARD) statement checklist was used for reporting [6].

### Study setting and population

This study was conducted in the ED of the University Medical Centre in Groningen, the Netherlands, a tertiary trauma centre receiving around 900 patients with a foot or ankle trauma per year. Patients presenting to the ED with a foot or ankle trauma were assessed according to standard clinical protocol. The triage nurses were trained to apply and document the OAR (Table 1). If positive, the patients were screened for eligibility [7].

**Table 1:** Ottawa Ankle Rules [3].

A series of Xrays films of the ankle and foot is required if:	
1. Patient not able to walk 4 steps or more, directly after trauma or in the examining room	
2. Bone tenderness posterior edge lateral malleolus (most distal 6 cm)	
3. Bone tenderness posterior edge medial malleolus (most distal 6 cm)	
4. Bone tenderness base fifth metatarsal bone	
5. Bone tenderness navicular bone	

### Inclusion and exclusion criteria

**Fig. 1:**
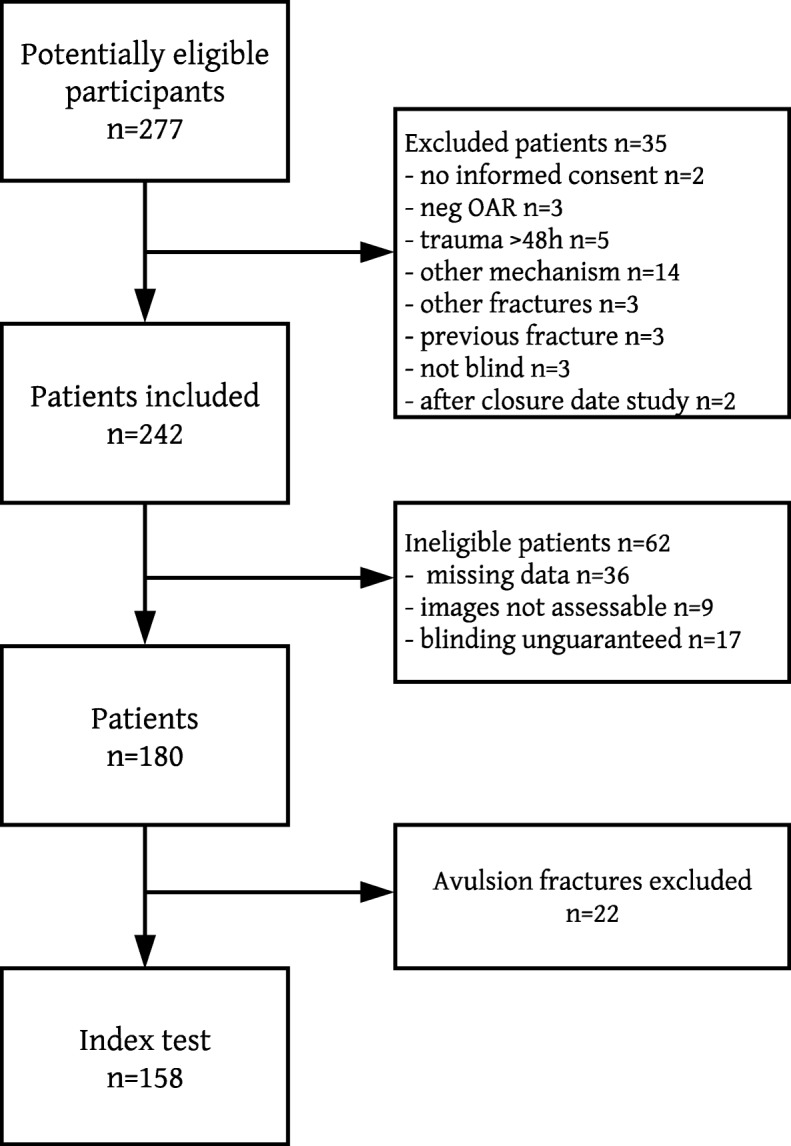
The process of inclusion, with the eligible, excluded and ineligible patients and avulsion fractures

Inclusion criteria were age ≥ 17 years, inversion or eversion trauma mechanism and a positive OAR. Exclusion criteria were negative OAR, open fractures, visible major dislocation, multi-trauma patients, previous fracture of the same ankle, degenerative ankle disease and a trauma ≥48 h, no informed consent, other fractures (calcaneus, tertiary malleolus), no blinding for the specific outcome of the OAR and the trauma mechanism, included after the closing date of the study [1, 5, 8–10]. Patients of whom the records were not evaluable were ineligible, avulsion fractures were excluded. (Fig. 1).

There was inclusion of consecutive patients, done by all the emergency physicians (EP’s) or residents, as in usual clinical practice. Informed consent and PoCUS were performed by an EP or resident. The sonographer was blinded for the trauma mechanism and the specific outcome of the OAR, by not knowing specifically which rules of the OAR led to a positive finding. The patient would consecutively be assessed and treated, in accordance with current practice, by another ED doctor blinded for PoCUS outcome. Radiography of the ankle included anteroposterior and lateral radiographs of the ankle, and fifth metatarsal bone if appropriate.

### Sonographers

All of the sonographers were either EP’s or residents with experience in PoCUS, trained with standardized sonography courses in emergency medicine. In total 23 sonographers were part of this study. These were 11 EP’s, 12 residents, and 1 EP expert sonographer. The expert sonographer was trained within the Ultrasound Leadership Academy Fellowship, a 12-month comprehensive course of PoCUS [11]. All sonographers were additionally trained by the expert in a 2-h theoretical and practical training specifically for PoCUS of the fifth metatarsal bone, distal tibia and fibula.

### Study protocol

PoCUS was performed initially on a Zonare Z One ultrasound machine, but after mechanical failure a SonoSite X-porte was used. A 10-MHz linear probe was used for sonography of 3 regions of the ankle:

1. Up to 10 cm proximal of the distal tibia.
2. Up to 10 cm proximal of the distal fibula.
3. Up to 5 cm proximal of the distal fifth metatarsal bone.

**Fig. 2:**
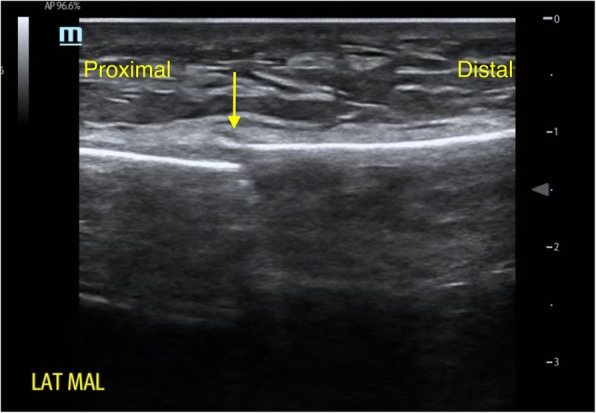
Ultrasound image of the lateral malleolus (= lat mal). The arrow points at cortical disruption

PoCUS was focussed on cortical disruption, indicating a fracture. PoCUS was not used to identify soft tissue or syndesmotic injuries of the ankle. The navicular bone was not scanned in this study with regards to low diagnostic value in previous research [10]. A 10-s sonography video of all patients was recorded to visualize the area of interest. Criteria for diagnosis of a fracture with PoCUS were cortical disruption or axial deviation of the bone surface as observed by the sonographer. See Fig. 2 for an example of a cortical disruption as seen by PoCUS. Fractures < 3 mm in width were considered non-significant avulsion fractures, as per previous studies [5].

All videos were secondarily assessed by the expert. Any differences between sonographers and expert sonographer were documented. The final evaluation of the radiographic images by the radiologist was considered the golden standard for the diagnosis of a fracture. The radiologist was blinded for the outcome of the PoCUS, but not for clinical findings that were documented. The result of the reference standard was not available to the sonographers or expert.

### Data analysis and sample size calculation

A sample size was calculated, using criteria based on previous research and desired study characteristics; a prevalence of patients with a fracture of 25%, a minimal acceptable sensitivity of 96% and minimal acceptable specificity of 97%, with a confidence interval of 5% for sensitivity. The calculated sample size for the determined sensitivity was 236 and for the specificity 62 patients [12].

Data analysis was done in SPSS Statistics 23 (IBM, Armonk, New York, USA). All demographics and clinical data were imported. Comparison between categorical variables was calculated by the chi-square test.

The measures of diagnostic accuracy were sensitivity and specificity of PoCUS for detection of fractures of the lateral and medial malleoli and fifth metatarsal bone compared to the golden standard radiographic images. Distal avulsion fractures of the fibula were not considered as a clinically relevant fracture, but mentioned [1].

## Results

A total of 277 patients could be assessed for eligibility. In total 242 were included, of which 62 ineligibles. The records of these ineligible patients were not evaluable due to missing data, non-assessable videos or because blinding was not guaranteed (thus when patients were included by the expert sonographer). Of the remaining 180 patients, 22 patients with avulsion fractures were excluded, leaving 158 patients for analysis, see Fig. 1. Baseline criteria and results are presented in Tables 2 and 3.

**Table 2:** Baseline criteria, including non-significant fractures

	Patients (n)	Percentage
**Total**	158	100%
**Demographics**		
Male	61	39%
Female	97	61%
Age (median)	28 (21–46)	
**Mechanism of trauma**		
Inversion	103	65%
Eversion	10	6%
Unknown	45	29%
**Activities**		
Walking	79	50%
Sports	54	34%
Traffic accident	16	10%
Other	3	2%
**OAR**		
Rule 1 (4 steps walking test)	117	74%
Rule 2 (lateral malleolus)	111	70%
Rule 3 (medial malleolus)	69	44%
Rule 4 (fifth metatarsal bone)	54	34%
Rule 5 (navicular)	44	28%
**PoCUS fracture sonographer**		
No fracture	118	75%
Fracture	40	25%
Distal tibia	2	1%
Distal fibula	28	18%
Fifth metatarsal bone	5	3%
Distal fibula and tibia	5	3%
Other	0	0%
**PoCUS fracture expert**		
No fracture	128	81%
Fracture	30	19%
Distal tibia	1	1%
Distal fibula	20	13%
Fifth metatarsal bone	5	3%
Distal fibula and tibia	4	3%
Other	0	0%
**Xray fracture**		
No fracture	123	78%
Fracture	35	22%
Distal tibia	0	0%
Distal fibula	20	13%
Fifth metatarsal bone	7	5%
Distal fibula and tibia	6	4%
Other^a^	2	1%

*OAR* Ottawa Ankle Rules, *PoCUS* point-of-care bedside ultrasound, *Xray* plain radiographic image. ^a^Other = malleolus tertius and distal tibia with malleolus tertius

**Table 3:** Clinically relevant fractures seen on radiography compared to point-of-care bedside ultrasound and sensitivity, specificity, positive predictive value (PPV) and negative predictive value (NPV) of the sonographers and expert in significant fractures

	Sonographer		Expert	
	Fracture	No Fracture	Fracture	No Fracture
**Fracture Xray**	28 (80%)	7 (20%)	29 (83%)	6 (17%)
**No fracture Xray**	12 (10%)	111 (90%)	1 (1%)	122 (99%)
**Sensitivity**	80.0% (95% CI 63.0–91.6%)		82.8% (95% CI 66.3–93.4%)	
**Specificity**	90.3% (95% CI 83.7–94.9%)		99.2% (95% CI 95.5–99.9%)	
**PPV**	70.0% (95% CI 57.0–80.3%)		96.7% (95% CI 80.3–99.5%)	
**NPV**	94.1% (95% CI 89.1–96.9%)		95.3% (95% CI 91.0–98.2%)	

In 35 patients (22%) significant fractures were seen using radiography. The sonographers and the expert identified 28 and 29 fractures, and missed 7 and 6 fractures, respectively. (see Tables 3, 4).

**Table 4:** Demographics of the nine missed fractures

Age	gender	Trauma mechanism	Activity	Fracture	Sonographer no	Sonographer seen	Expert seen
24	male	Inversion	sports	MT V	1	no	yes
18	female	Inversion	walking	MT V	2	no	yes
23	female	Unknown	walking	MT V (Jones)	4	no	no
57	female	Unknown	walking	distal fibula	3	no	no
55	female	Unknown	traffic accident	MT V	3	no	no
19	female	Eversion	traffic accident	distal tibia + malleolus tertius	7	no	yes
19	female	Unknown	traffic accident	distal fibula	5	no	no
23	female	Eversion	walking	distal fibula	6	yes	no
19	male	Unknown	sports	distal fibula	8	yes	no

*MTV* fifth metatarsal bone

The sensitivity of PoCUS in detecting clinically significant fractures by all sonographers was 80.0% (95% Confidence Interval (CI) 63.0 to 91.6%), specificity 90.3% (95% CI 83.7 to 94.9%), positive predictive value 70.0% (95% CI 57.0 to 80.3%) and the negative predictive value 94.1% (95% CI 89.1 to 96.9%). The sensitivity of PoCUS in detecting clinically significant fractures by the expert was 82.8% (95% CI 66.3 to 93.4%), specificity 99.2% (95% CI 95.5 to 99.9%), positive predictive value 96.7% (95% CI 80.3 to 99.5%) and the negative predictive value 95.3% (95% CI 91.0 to 98.2%), see Table 3.

There were no adverse events from performing PoCUS or the reference standard, radiography.

## Discussion

This prospective cohort study, which is the most relatable to usual clinical practice so far, found PoCUS to have a high specificity and negative predictive value in diagnosing significant fractures of the ankle and foot [9].

PoCUS performed in consecutive patients by all sonographers, with different levels of experience, results in a sensitivity of 80.0% and specificity of 90.3%. This is less compared to previous studies [8, 10]. However, these studies were not blinded and used only 1–5 sonographers, which is not realistic in usual clinical practice. In common clinical practice, sonography will be performed by both EP’s as well as residents, with a difference in experience in PoCUS. This usual clinical practice is well reflected in our study, where the experience amongst the 23 sonographers varied in PoCUS for different uses and specifically this indication, as well as the exposure to foot and ankle trauma in our tertiary centre.

For this study, sonographers were blinded for the history and, as far as possible, abnormalities shown in the physical examination and positive rules of the OAR. This was the most valid way to study sensitivity and specificity of PoCUS only, without bias. In usual clinical practice, PoCUS would be executed by the same person taking the history and performing the physical examination. Thus, combining clinical knowledge with the abnormalities seen with PoCUS may very well further improve the diagnostic value of this test.

The results of the expert in our study show that more experience with PoCUS for this indication and frequent exposure will improve diagnostic value. Also, PoCUS is a dynamic examination, and when assessing an exam performed by someone else, interpretation might be different, which explains the missed fractures of the expert. However, when compared to previous studies, the expert had comparable diagnostic value, even whilst being blinded for the history and clinical examination [8, 10].

It appears that, using PoCUS in acute foot and ankle trauma, albeit a high negative predictive value, there is a risk of missing fractures and only a minimal risk for false positive fractures. When instructing the patient, who has a negative PoCUS exam on presentation, to return for additional assessment if complaints persist or aggravate, missed fractures can be intercepted and treated accordingly.

This sensitivity corresponds to the 12 false positive fractures (10%) diagnosed by the sonographers. False positive fractures would result in treating the patient avoidably as a fracture, with a backslap, which is also an accepted treatment for sprain. In this case usual clinical practice would be to assess the patient clinically within a week, which could prevent longer immobilisation.

In this study an overall 76% absence of fractures, confirmed with radiography, was comparable to known results of the OAR [4]. If patients were assessed with PoCUS by a sonographer in combination with the OAR alone, radiographic imaging could have been prevented in 80%, the amount of rightly diagnosed non-fractures. The high negative predictive value of the test supports these findings. This is in accordance with previous studies, which also show a reduction of radiological assessment of approximately 80% [2, 5].

Patients with negative OAR and PoCUS, could have been treated for sprain or avulsion fracture in the prehospital setting without presentation to the ED. With the current increase in crowding in the ED, PoCUS for pre-hospital or general practitioner triage could be beneficial. This accounts for outpatient clinics and the developing world (where radiology is expensive), as recommended by The World Health Organization [1]. Besides this, portable handheld PoCUS is already available and might be implemented in future hospital settings, making quick bedside diagnostics accessible.

### Limitations

This study was conducted in an academic tertiary hospital, which may result in bias especially for results in primary or secondary care. There were more ineligible patients than expected, due to missing data, non-assessable videos or because blinding was not guaranteed (thus when patients were included by the expert sonographer). The missing data caused a certain degree of bias. During the study period the ultrasound machine un-expectantly broke down resulting in a temporary pause in inclusion and images that could not be saved correctly or were lost in the process. Total blinding could not always be assured because of visible hematoma and swelling in foot and ankle trauma, however, this reflects common practice. In young patients under 20 years, the epiphyseal plate could still be present, in which case the hypoechoic appearance of the epiphyseal cartilage might appear as a cortical disruption when PoCUS is being performed by less experienced sonographers. However, there is a small probability of a still existing epiphyseal plate in the number of patients ≥17 years and under 20 years in this study.

## Conclusion

PoCUS combined with the OAR has a good diagnostic value in usual clinical practice in the assessment of suspected ankle and fifth metatarsal bone fractures compared to radiographic imaging. In this study, we show that more experience with PoCUS and frequent exposure will further improve diagnostic value. Implementing PoCUS for the evaluation of ankle and fifth metatarsal bone trauma in the ED can possibly reduce the use of radiography and minimize the exposure to radiation, time, costs and burdening of the patient and ED.

## Data Availability

Dataset will not be online available because individual privacy could be compromised. The dataset is available and archived as electronic file in the emergency department of the UMCG.

## Abbreviations

ED: Emergency Department
EP: Emergency physician
OAR: Ottawa Ankle Rules
PoCUS: Point-of-care bedside ultrasound
STARD: Standards for Reporting Diagnostic Accuracy
CI: Confidence interval
